# Difference Analysis of Gas Molecules Diffusion Behavior in Natural Ester and Mineral Oil Based on Molecular Dynamic Simulation

**DOI:** 10.3390/molecules24244463

**Published:** 2019-12-05

**Authors:** Wenyu Ye, Jian Hao, Yufeng Chen, Mengzhao Zhu, Zhen Pan, Fei Hou

**Affiliations:** 1 School of Electrical and Electronic Engineering, Shandong University of Technology, Zibo 255000, China; feitianxiexian@126.com (Z.P.); hff941101@163.com (F.H.); 2 State Key Laboratory of Power Transmission Equipment & System Security and New Technology, Chongqing University, Chongqing 400044, China; haojian2016@cqu.edu.cn; 3 Shandong Electric Power Research Institute, State Grid Shandong Electric Power Co., Jinan 250003, China; MycyPost@163.com (Y.C.); xiaozhupost@163.com (M.Z.)

**Keywords:** natural ester, gas molecules, molecular dynamics, diffusion, mean square displacement

## Abstract

Natural ester, as a new environmentally green insulating oil, has been widely used in transformer. In an oil-immersed transformer, the normal aging, thermal failure, and discharge failure could easily lead to the decomposition of the oil-paper insulation system and produce different kinds of gases. Studying gas dissolution in natural ester and mineral oil could provide assistance in applying criteria to make a diagnosis of different kinds of faults in the transformer. In this paper, the molecular dynamics method was used to investigate the diffusion behavior of seven fault characteristic gases (including H_2_, CO, CH_4_, C_2_H_2_, CO_2_, C_2_H_4_, C_2_H_6_) in natural ester and mineral oil. The simulation parameters of free volume, interaction energy, mean square displacement, and diffusion coefficient were compared between the natural ester and mineral oil. Meanwhile, the influence of temperature on the diffusion of gas molecules in two kinds of oils was also analyzed. Results showed that the free volume, the interaction energy, and the relative molecular mass of gas molecules were the factors influenced by the diffusion of gas molecules in natural ester and mineral oil. The order of the diffusion coefficients of gas molecules in natural ester was as follows: H_2_ > CH_4_ > CO > C_2_H_2_ > C_2_H_4_ > CO_2_ > C_2_H_6_ and that in mineral oil was as follows: H_2_ > CH_4_ > CO> C_2_H_2_ > C_2_ H_4_ > C_2_H_6_ > CO_2_. By comparing the diffusion behavior of gas molecules in natural ester and mineral oil, it was found that the smaller free volume and higher interaction energy of gas molecules in natural ester were the major reasons for the gas molecules to be more difficult to diffuse in natural ester. The rising temperature could enhance the free volume and reduce the interaction energy between gas molecules and oil. The diffusion coefficient of gas molecules increased exponentially with the follow of temperature. However, the temperature didn’t affect the ordering of diffusion coefficient, free volume, and interaction energy of gas molecules in natural ester and mineral oil.

## 1. Introduction

One of the most important electrical equipment in an electrical power system is a transformer, and it plays a significant role in providing an efficient and reliable electricity supply. The oil-paper insulation system is used as the main insulation structure in transformers. Because of its economic performance and good insulation characteristics, mineral oil has been extensively used in transformers. However, some disadvantages of mineral oil, such as low flash point, poor biodegradation rate, and non-renewability, would cause a large number of troubles. Therefore, an increasing number of scholars keep their eyes on the insulation properties of natural ester oils and their applications [1,2]. The benefits of natural ester oils contain increased fire safety, higher moisture tolerance, and reduction of cellulose degradation [3,4,5]. Meanwhile, natural ester oil is found to be suitable to be used in transformers [6,7].

The oil-immersed transformer is inevitably affected by thermal, electric, and mechanical forces in operation, which leads to the failure of the oil-paper insulation system. At the same time, these faults produce small gas molecules, such as H_2_, CO_2_, CO, etc., which dissolve in the oil and diffuse as bubbles. Several scholars have studied the gas production characteristics of natural ester and mineral oil. Piotr Przybylek et al. analyzed the gas production characteristics of mineral oil, natural ester, and synthetic oil in electrical and thermal failure [8]. Zerye Ayalew et al. showed that the fault of natural ester and mineral oil mainly produced H_2_, CO, CH_4_, C_2_H_2_, CO_2_, C_2_H_4_, and C_2_H_6_ [9]. To determine how to combine the produced gas with fault analysis is a major problem.

The method that uses the dissolved gas in oil to diagnose the insulation status of transformers first appeared in the 1960s. With the subsequent research and development, IEC 60599-1999 indicates that the insulation status of transformers can be diagnosed by the content of dissolved gas in mineral oil. IEEE C57.155-2014 guide for interpretation of gases generated in natural ester and synthetic ester-immersed transformers assists the transformer operator in evaluating dissolved gas analysis (DGA) data obtained from natural ester, providing convenience for the status diagnosis of oil-paper insulation system. However, little work has been focused on the mechanism of gas diffusion in natural ester and mineral oil and the effect of temperature in the DGA. The diffusion of gas in oil depends on the type of gas, the type of oil, and the diffusion temperature. The diffusion behavior of different gases and equilibrium time are different. If the gas does not diffuse to the location of the oil sample extraction during the equilibrium time, then the fault diagnosis result is inaccurate. As the composition of mineral oil is different from that of natural ester, it is necessary to study and compare the diffusion behavior of gas molecules in natural ester and mineral oil.

During the past decade, the theory of computer science and computational algorithms has developed in a variety of directions, and the study of molecular dynamics simulation technology in the field of transformer oil-paper insulation has been greatly matured. There are many reports about the application of molecular dynamics simulation technology in the oil-paper insulation system. The diffusion of small molecular gases in mineral oil and cellulose was studied by molecular simulation in reference [10]; nevertheless, the model size, simulation time, and simulation parameters could be further optimized to improve the accuracy of the results. Comparing the results of reference [10], it could be found that the diffusion coefficient is an order of magnitude, but there are some differences in the order of diffusion of gas molecules. The diffusion of water molecules in the oil-paper insulation system was studied according to reference [11]. It has been shown that cellulose and oil have adsorption effects on water molecules, and the interaction between water molecules and cellulose is stronger. The interaction energy between mineral oil and cellulose was studied by molecular simulation in reference [12]. The results showed that the interaction between mineral oil and fiber was different, and the hydroxyl group was an important factor affecting the interaction between cellulose and mineral oil. Mengzhao Zhu et al. studied the diffusion of hydronium ions in mineral oil by simulation, and molecular dynamics simulation indicated that the water content in oil had a great influence on the diffusion of hydronium ions [13]. Materials Studio software was used to simulate the interaction between the nanoparticles and cellulose molecules in reference [14]. Molecular dynamic, as a new research method, is helpful to study the diffusion behavior of gas molecules in different insulating oils from the microscopic perspective.

In this paper, to reveal the diffusion mechanism of gases in natural ester and mineral oil, the diffusion behavior of seven kinds of gas molecules in natural ester and mineral oil was studied by using molecular dynamics simulation technique. Firstly, Material Studio software (BIOVIA, San Diego, CA, USA) was used to build two groups of models to simulate the diffusion of gas molecules. Then, the diffusion of gas molecules in natural ester and mineral oil was compared, and their mechanism was analyzed. Finally, the influence of temperature on the diffusion of gas molecules in natural ester and mineral oil was analyzed by simulation from 283 K to 363 K. Moreover, this work provided assistance in understanding and applying criteria and the real-time diagnosis of faults.

## 2. Molecular Dynamics Simulation

### 2.1. Model Setup

**Figure 1:**
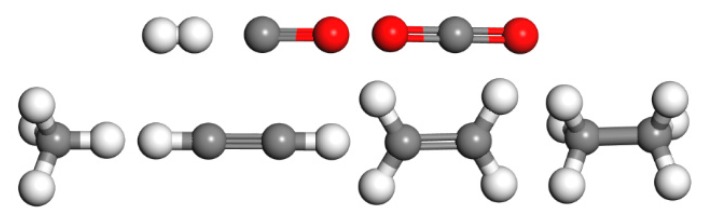
Gas molecular models. The gas molecules are H_2_, CO, CO_2_, CH_4_, C_2_H_2_, C_2_H_4_, and C_2_H_6_.

The main gases molecules in mineral oil and natural ester are as follows: hydrocarbons, such as methane (CH_4_), acetylene (C_2_H_2_), ethylene (C_2_H_4_), and ethane (C_2_H_6_), carbon oxides, such as carbon monoxide (CO) and carbon dioxide (CO_2_), and hydrogen (H_2_). The gas molecular models are shown in Figure 1.

The natural ester model was built on the basis of soybean natural ester. Each natural ester model was composed of 10 triglyceride molecules. The saturated fatty acid in soybean oil-based natural ester is stearic acid (C_18:0_, CH_3_(CH_2_)_16_COOH), the monounsaturated fatty acid is mainly oleic acids (C_18:1_, CH_3_(CH_2_)_7_CH=CH(CH_2_)_7_COOH), the unsaturated fatty acid (double bonds) is mainly linoleic acid (C_18:2_, CH_3_(CH_2_)_4_CH=CHCH_2_CH=CH(CH_2_)_7_COOH), and the unsaturated fatty acid (triple bonds) is linolenic acid (C_18:3_, CH_3_CH_2_CH=CHCH_2_CH=CHCH_2_CH=CH(CH_2_)_7_COOH). The main fatty acid compositions of natural ester are shown in Table 1.

The mineral oil model was established based on cycloalkyl mineral oil [11]. The basic physics and chemical properties of cycloalkyl mineral oil are mainly determined by chain hydrocarbon and naphthenic hydrocarbon, according to mass spectrometry. The main component mass fractions of mineral oil are shown in Table 2.

**Figure 2:**
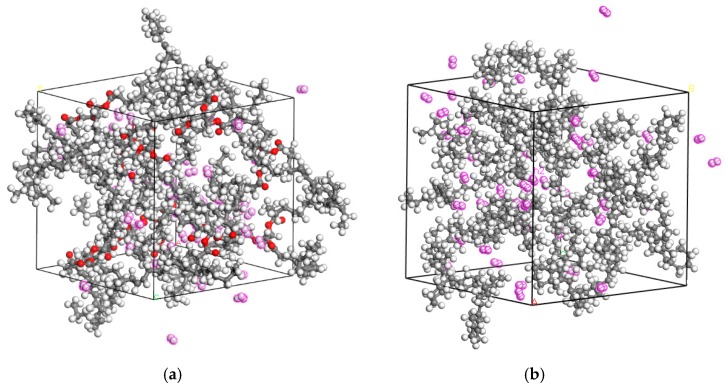
H_2_ molecular models in oil. (**a**) Model diagram of H_2_ molecular models in natural ester. (**b**) Model diagram of H_2_ molecular models in mineral oil. The one circled by the pink line is H_2_.

Gas within natural ester models and mineral oil models were established, respectively. The number of gas molecules did not affect the final simulation result. If the number of gas molecules is too small, the result will be more accidental. Accordingly, in each model, 45 gas molecules were added. Taking the diffusion model of H_2_ molecules in mineral oil and natural ester as an example, the constructed model is shown in Figure 2.

### 2.2. Simulation Processes and Parameters

The amorphous cell tools module was used to build the model, and each model was geometrically optimized. The initial energy of each model was very high. High temperature can provide more energy to the system in a shorter time in order to overcome the energy barrier and find the lowest point of energy in molecular dynamics simulation. Therefore, to make the model reasonable, geometry and energy optimization was required. The treatment process included structural refinement, volume relaxation, and annealing. Firstly, in the process of structural optimization, the default smart algorithm was adopted, which meant a rough optimization by the steepest descent method followed by a further optimization in the conjugate gradient method with 5000 steps. Then, during the annealing, the temperature started from 300 to 1000 K. The annealing time was set at 100 ps, and the energy minimization was carried out for every annealing step. After the above treatment, the molecular dynamics simulation was carried out in the next stage. Constant-pressure and constant-temperature (NPT) ensemble, with a constant number of molecules, pressure, and temperature, was used to balance each model with 500 ps. Then, the canonical ensemble (NVT) ensemble, with a constant number of molecules, volume, and temperature, was used for molecular dynamics simulation with 500 ps. Each model was simulated during heating from 283 K to 363 K, with the simulation results recorded every 20 K.

Molecular dynamics simulation was performed by the pcff force field, which has been proved to be applicable to carbohydrate calculations [10,11]. The atom-based Ewald method was used in the calculation of the Van der Waals and electrostatic action. The nose method was used to control temperature [15]. Berendsen method was used to control pressure [16], and the pressure was set to 101.3 kPa. Materials Studio software was used for the whole simulation process, and part of the data was collected by self-scripted.

### 2.3. Calculation Method

#### 2.3.1. Free Volume

The free volume of gas molecules in oil is an important factor influencing the diffusion behavior of gas molecules. Based on Fox and Flory’s free volume theory [17], the occupied volume (*V_O_*) and the free volume (*V_F_*) constitute the total volume (*V_T_*) of the polymer according to Equation (1). The free volume is scattered in the whole polymer in the form of the void, and it is because of the free volume that the molecules in the polymer are in motion. The fraction of free volume (*FFV*) is the ratio of the free volume to the total volume. Because of the relativity of free volume, the free volume of different gas molecules in the same medium is different. The free volume of gas molecules is determined by the nature and size of gas molecules. During the simulation, atom volume and the surface tool was used to calculate the free volume of seven gas molecules under the surface of Connolly.

(1) $$FFV=\frac{V_{F}}{V_{F}+V_{O}}$$

#### 2.3.2. Interaction Energy

There is interaction energy between the gas molecules and the mineral oil, which plays a significant role in gas diffusion behavior. The interaction energy between substances in the simulation model can be obtained by Equation (2):

(2) $$E_{\mathrm{int}}=E_{t}-\left(E_{A}+E_{B}\right)$$

where *E_int_* is the interaction energy between the two substances in the model, *E_t_* is the total energy in the model, *E_A_* and *E_B_* are the energies of A and B. The interaction is positive, which means the two substances repel each other. Contrarily, it means that two substances are attracted to each other. The bigger the negative, the more attractive. The interaction energy mainly consists of Van der Waals interaction energy and electrostatic interaction energy. The Van der Waals interaction energy can be expressed as follows:

(3) $$E_{\mathrm{vdw}}=D_{0}\left[2{\left(\frac{R_{0}}{R}\right)}^{9}-3{\left(\frac{R_{0}}{R}\right)}^{6}\right]$$

where *E*_vdw_ is the Van der Waals interaction energy, *D_0_* is equilibrium well depth, *R_0_* is equilibrium distance, and *R* is the distance between two particles. The electrostatic action energy can be calculated as follows:

(4) $$E_{\mathrm{elec}}=C\frac{q_{i}q_{j}}{\varepsilon R}$$

where *E*_elec_ is the electrostatic interaction energy, c is a unit conversion factor, ε is relative dielectric constant, *R* is the distance between two particles, and q is the charge of particles.

#### 2.3.3. Diffusion Displacement

The displacement of gas molecules can well reflect the diffusion behavior of gas molecules, and it can be calculated, as shown in Equation (5).

(5) $$R(t)=\sqrt{{\left|\left(r(t)-r(0\right)\right|}^{2}}$$

where *R(t)* is the displacement of the water molecule relative to the initial moment at time t. *r(t)* and *r(0)*, respectively, represent the coordinates of the water molecule at time t and time 0.

#### 2.3.4. Mean Square Displacement

The motion state of gas molecules can be expressed by mean square displacement (MSD), which describes the average distance of all particles from their initial point at time t. MSD can be expressed as the following equation:

(6) $$MSD=\langle |\overset{}{\vec{r_{i}}(t)-}{\vec{r_{i}}(0)|}^{2}\rangle$$

where ${\vec{r}}_{i}\left(t\right)$ and ${\vec{r}}_{i}\left(0\right)$ represent the position vector of the atom at time t and time 0, respectively. < > represent the average of all particles in the model. The diffusion coefficient is an important parameter to characterize the diffusion ability of matter, which can be solved by Einstein formula. The diffusion coefficient can be derived from Equation (7) [18].

(7) $$D=\frac{{\left|\vec{r}(t)-\vec{r}(0)\right|}^{2}}{6t}=\frac{a}{6}$$

where a is the slope of the curve fitted by MSD.

#### 2.3.5. Correlation Analysis

The correlation between different factors and diffusion can be calculated by the Pearson correlation coefficient. Pearson correlation coefficient can be obtained from the following Equation (8):

(8) $$r=\frac{\sum x_{i}y_{i}-\frac{\sum x_{i}\cdot \sum y_{i}}{n}}{\sqrt{\left({\sum x_{i}}^{2}-\frac{{\left(\sum x_{i}\right)}^{2}}{n})(\sum y_{i}{}^{2}-\frac{{\left(\sum y_{i}\right)}^{2}}{n}\right)}}$$

where *r* is the Pearson correlation, which is between negative 1 and 1. When r is greater than 0, two variables are positively correlated. On the contrary, two variables are negatively correlated. Data correlation can be divided into three levels. Additionally, when the absolute value of r is less than 0.4, it indicates a low degree of linear correlation; when the absolute value of r is greater than 0.4 and less than 0.7, it indicates a significant correlation; when the absolute value of r is greater than 0.7, it indicates a high degree of linear correlation.

## 3. Results and Discussion

### 3.1. Analysis of Factors and Differences Affecting Diffusion

#### 3.1.1. Free Volume Analysis

**Figure 3:**
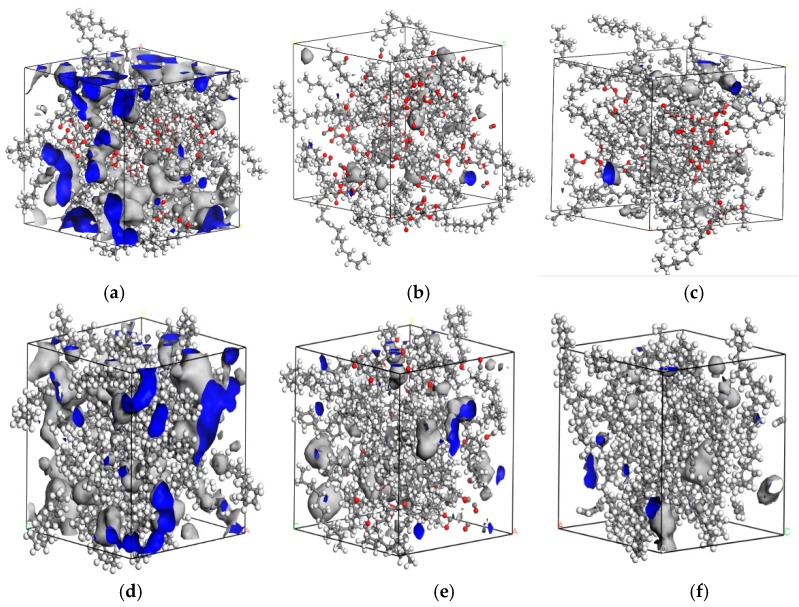
The volume of gas molecules. The volume of (**a**) H_2_, (**b**) CO, and (**c**) C_2_H_2_ in natural ester. The volume of (**d**) H_2_, (**e**) CO, and (**f**) C_2_H_2_ in mineral oil.

The free volume of gas molecules in oil is one of the important factors affecting the diffusion of gas molecules. Figure 3 demonstrates the simulation results of part of gas molecules by giving the detail of cell volume in gas molecules with natural ester and mineral oil at 343 K. The grey part shows the occupied volume, and the blue one stands for free volume. The fractional free volume of seven gas molecules in natural ester with 343 K was calculated, as shown in Table 3. The fractional free volume of H_2_ was the largest, and the free volume of C_2_H_6_ was the smallest; 12.29 and 3.45, respectively. The *FFV* order of gas molecules in natural ester was as follows: H_2_ > CO > CH_4_ > C_2_H_2_> CO_2_ > C_2_H_4_ > C_2_H_6_.

**Figure 4:**
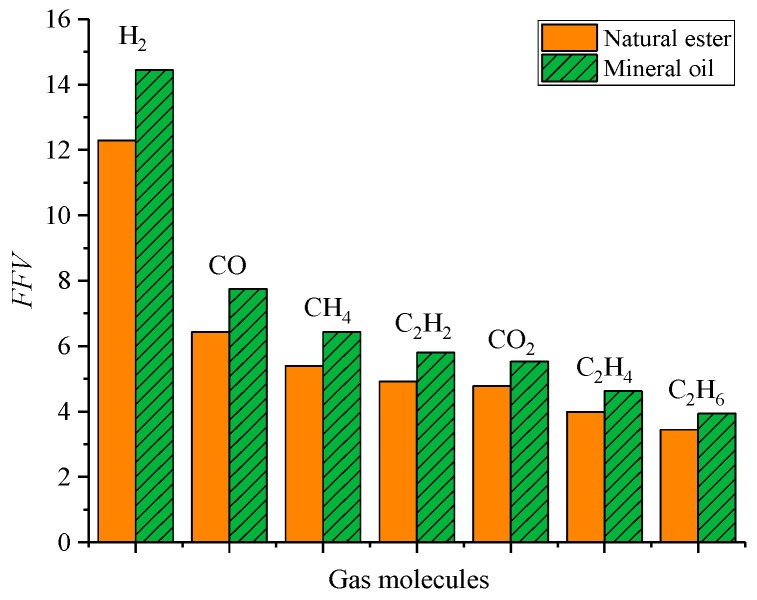
Comparison diagram of the fractional free volume of gas molecules in natural ester and mineral oil.

Compared with the natural ester, the sequence of *FFV* of gas molecules was consistent, as listed in Table 4 and Figure 4, which was related to the radius of gas molecules. However, the *FFV* of gas molecules in mineral oil was greater. It is well known that natural esters have a higher viscosity than mineral oils [19]. According to the free volume theory [20], the relationship between viscosity and free volume can be calculated by the following Equation (9):

(9) $$\ln n_{0}=\ln A+B(V-V_{f})/V_{f}$$

where n_0_ is the viscosity, V is total volume, *V_f_* is free volume, A and B are constants. It can be seen that the larger the viscosity, the smaller the free volume. Therefore, the *FFV* of gas molecules in natural ester was less than that in mineral oil.

#### 3.1.2. Interaction Energy Analysis

**Figure 5:**
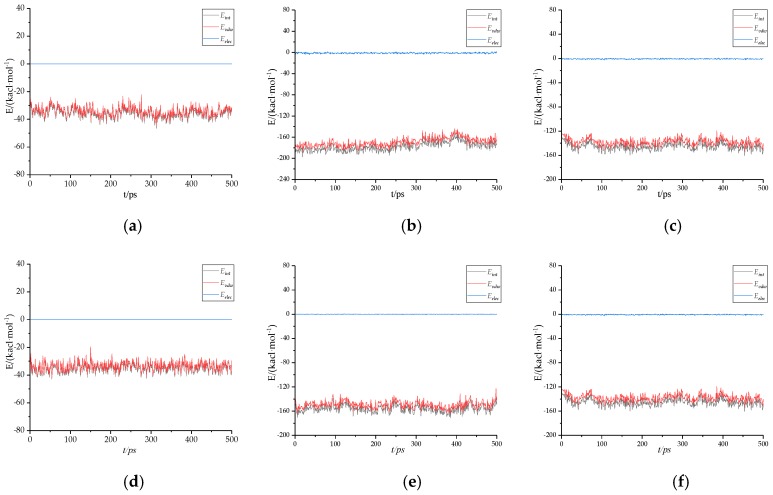
The interaction energy of gas molecules and oil varies with time at 343 K. The interaction energy between (**a**) H_2_, (**b**) CO, and (**c**) C_2_H_2_ and natural ester. The interaction energy between (**d**) H_2_, (**e**) CO, and (**f**) C_2_H_2_ and mineral oil. The black line is *E_int_,* the red line is *E_vdw_,* and the blue line is *E_elec_*. *E_int_* is the interaction energy between gas and oil, *E_vdw_* and *E_elec_,* respectively, represent the Van der Waals action energy and electrostatic action energy of gas molecules and oil.

The interaction energy between gas molecules and oil affected the diffusion behavior of gas molecules. Taking H_2_, CO, and C_2_H_2_ as examples, Figure 5 sketches the interaction energy of gas molecules, with natural ester and mineral oil changed over time. In Figure 5, *E_int_* is the interaction energy between gas and oil, *E_vdw_* and *E_elec_,* respectively, represent the Van der Waals action energy and electrostatic action energy of gas molecules and oil. It could be seen that the interaction energy between H_2_, including the remaining gas molecules, and oil could fluctuate up and down a certain value with the change of simulation time, meaning that each model had reached equilibrium, and the simulation results were reliable.

Table 5 shows average interaction energy between gas molecules and natural ester at 343 K. It could be seen that the interaction energy between the gas molecules and natural ester was the binding energy, the Van der Waals interaction energy was the main interaction energy, and the electrostatic interaction energy was extremely low. According to Table 5, the binding energy of H_2_ and natural ester was the smallest, only reaching 35.56 kcal/mol, the binding energy of hydrocarbon gas was 124.7~206.34 kcal/mol, and the binding energies of CO and CO_2_ had little difference, respectively, 177.51 kcal/mol and 168.34 kcal/mol. The interaction energy between gas molecules and natural ester was as following: C_2_H_6_ > C_2_H_4_ > CO > CO_2_ > C_2_H_2_ > CH_4_ > H_2_.

The average interaction energy between gas molecules and mineral oil at 343 K was calculated, as depicted in Table 6. Van der Waals energy was still the main interaction energy between gas molecules and mineral oil. The binding energy of H_2_ and mineral oil only reached 33.36 kcal/mol. The binding energy of hydrocarbon gas was 121.31~187.26 kcal/mol. The binding energies of CO and CO_2_ were almost the same, respectively, 155.8 kcal/mol and 158.1 kcal/mol. The interaction energy between gas molecules and mineral oil was as following: C_2_H_6_ > C_2_H_4_ > CO_2_ > CO > C_2_H_2_ > CH_4_ > H_2_, which was different from the sequence of interaction energies of gas molecules in natural esters.

**Figure 6:**
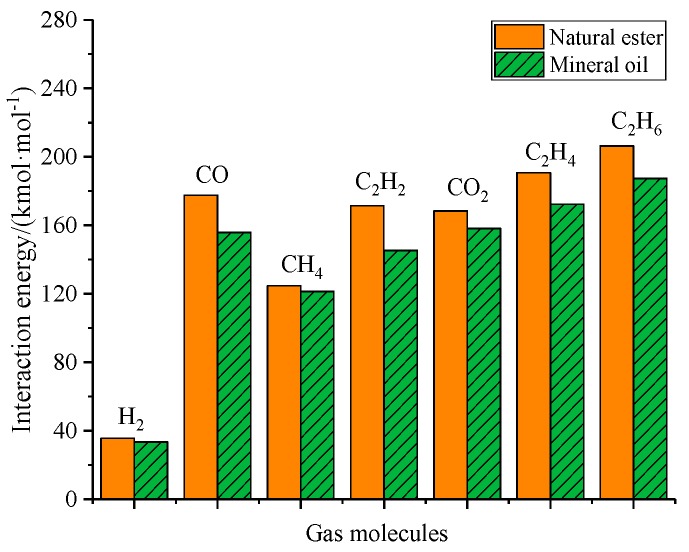
Comparison diagram of the interaction energy between gas molecules and oil.

Figure 6 describes the difference in the interaction energy of gas molecules between the natural ester and mineral oil. It was clear that the interaction energy between gas molecules and natural esters was generally higher than that between gas molecules and mineral oils. Among all gas molecules, the interaction energy difference of H2 was the smallest, only 2.2 kcal/mol, and the interaction energy difference of CO was the largest, up to 21.71 kcal/mol. This phenomenon could be attributed to the composition difference between the natural ester and mineral oil.

#### 3.1.3. Relative Molecular Weight Analysis

When comparing the diffusion behavior of different gas molecules, the relative molecular weight of the gas could not be ignored. Table 7 shows the relative molecular weight of gas molecules. By comparing the relative molecular weight and interaction energy of gas molecules, it could be found that the larger the molecular weight of gas molecules with similar structures, the greater the interaction energy. But CO and CO_2_ in natural esters were an exception.

### 3.2. Diffusion Displacement and Trajectory of Gas Molecules

**Figure 7:**
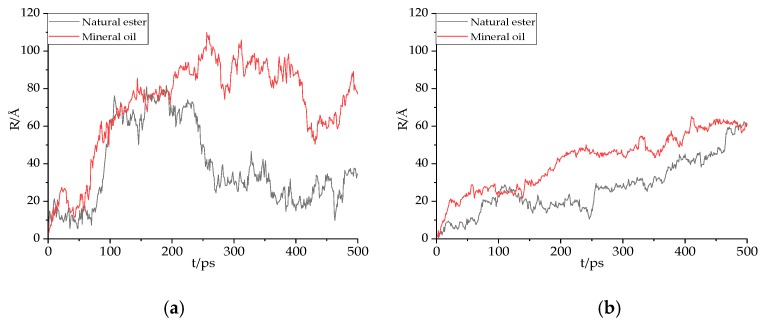

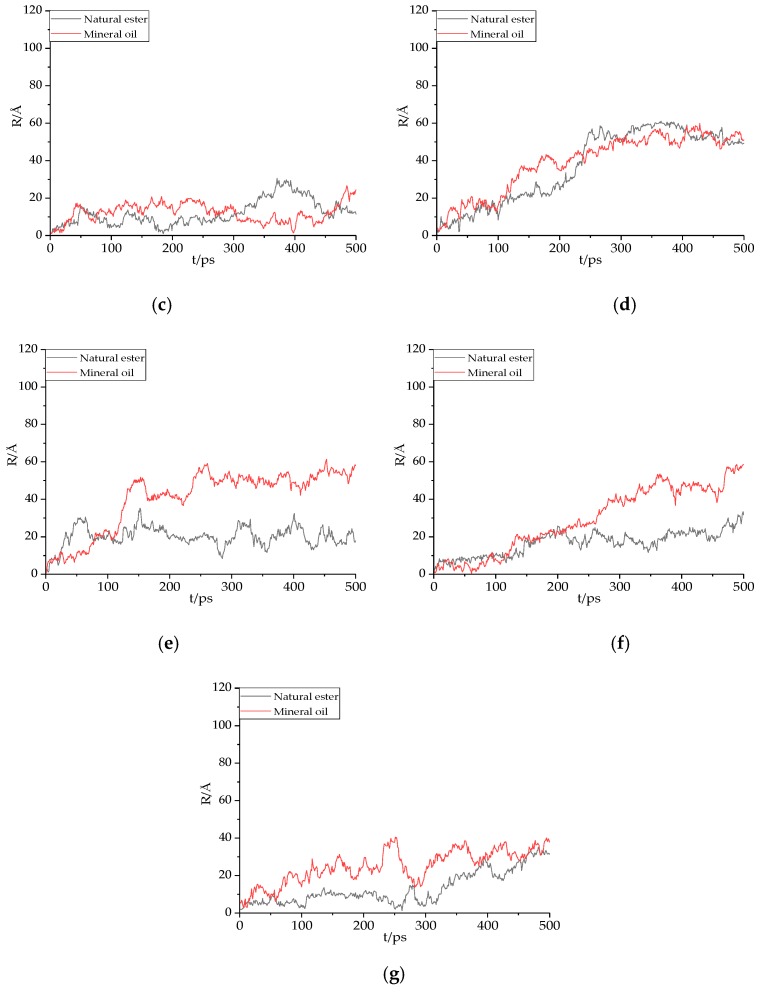
The displacement of gas molecules in oil at 343 K. (**a**) H2, (**b**) CO, (**c**) CO2, (**d**) CH4, (**e**) C2H2, (**f**) C2H4, and (**g**) C2H6 in natural ester and mineral oil. The black line is the displacement of gas molecules in natural esters, and the red line is the displacement of gas molecules in mineral oil.

The displacement of gas molecules in natural ester and mineral oil under 343 K is depicted in Figure 7. It was found that the displacement of the gas molecules in the oil was zigzag, indicating that the gas molecules diffuse in the form of continuous jumps. This result indicated that gas molecules could conduct continuous transformational diffusion in the holes of the medium, whether in natural ester or mineral oil. On the one hand, whether the gas molecules were in natural ester or mineral oil, H_2_ had the greatest displacement distance. H_2_ jumped a lot more each time than other gas molecules, making it easier to spread. On another hand, gas molecules tended to move closer in natural ester compared with mineral oil.

**Figure 8:**
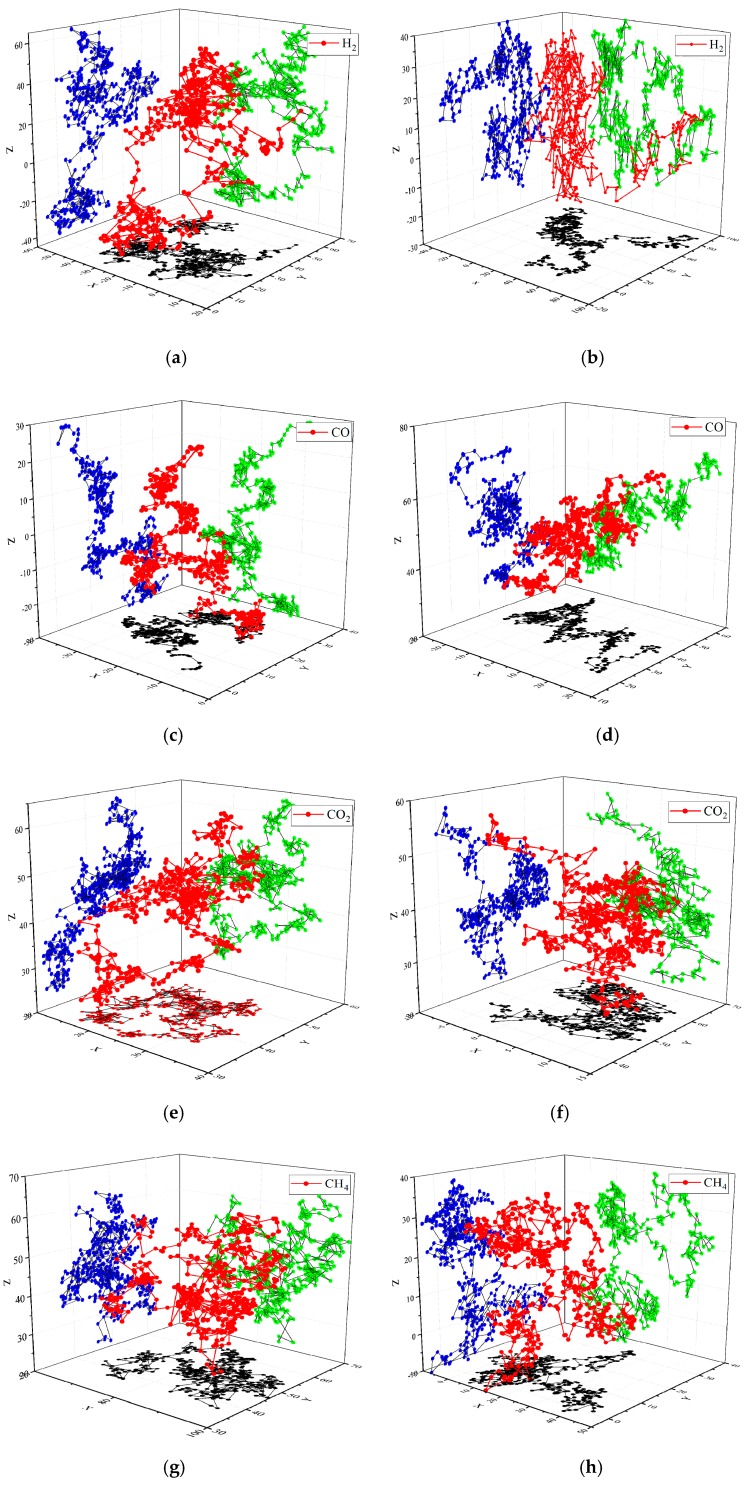

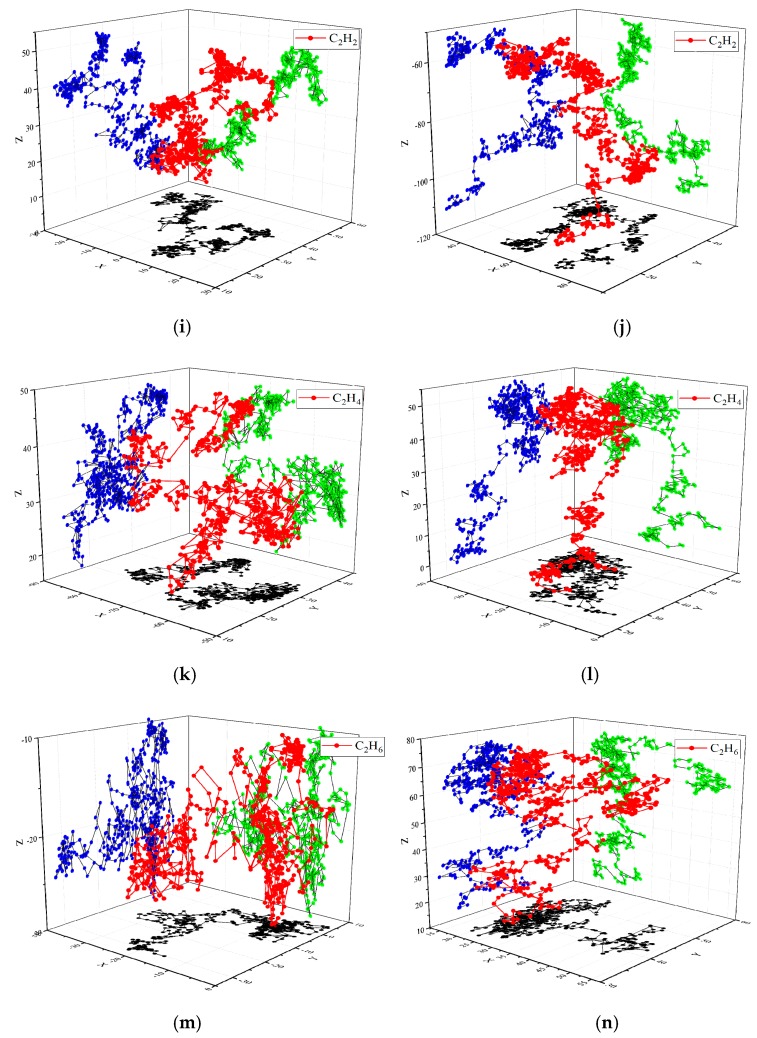
The trajectory of gas molecules in oil at 343 K. (**a**) H_2_, (**c**) CO, (**e**) CO_2_, (**g**) CH_4_, (**i**) C_2_H_2_, (**k**) C_2_H_4_, and (**m**) C_2_H_6_ in natural ester. (**b**) H_2_, (**d**) CO, (**f**) CO_2_, (**h**) CH_4_, (**j**) C_2_H_2_, (**l**) C_2_H_4_, and (**n**) C_2_H_6_ in mineral oil.

Figure 8 shows the diffusion trajectory of gas molecules in two kinds of oil. The lines formed by the red dots are the three-dimensional diffusion trajectories; the lines formed by the black dots, green dots, and the blue dots are the projections of the diffusion trajectories on the xy, xz, and yz planes, respectively. It could be seen that the diffusion trajectories in xx, xy, and xz planes were basically the same, the trajectory range of H_2_ was the largest, the diffusion trajectory was scattered, and the range of CO_2_ and C_2_H_6_ diffusion trajectories was small. The diffusion trajectories of the gas molecules in the two kinds of oils were compared, and it could be found that the diffusion trajectories of gas molecules in natural esters were smaller and denser than that in mineral oil.

### 3.3. Diffusion Coefficient and Correlation Analysis of Gas Molecules

**Figure 9:**
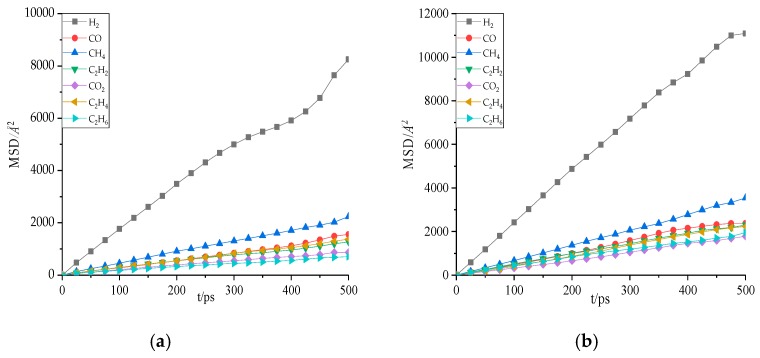
The mean square displacement (MSD) of gas molecules in oil at 343 K. (**a**) Gas molecules in natural ester. (**b**) Gas molecules in mineral oil.

The MSD curves of seven gas molecules in natural ester and mineral oil at 343 K is illustrated in Figure 9. It was distinctly observed that different gas molecules had different MSD curves. There was an evident difference in MSD between H_2_ molecules and the other six gas molecules. The MSD of H_2_ was largest in two kinds of oil, and the MSD of C_2_H_6_ and CO_2_ was lowest in natural ester and mineral oil, respectively.

**Figure 10:**
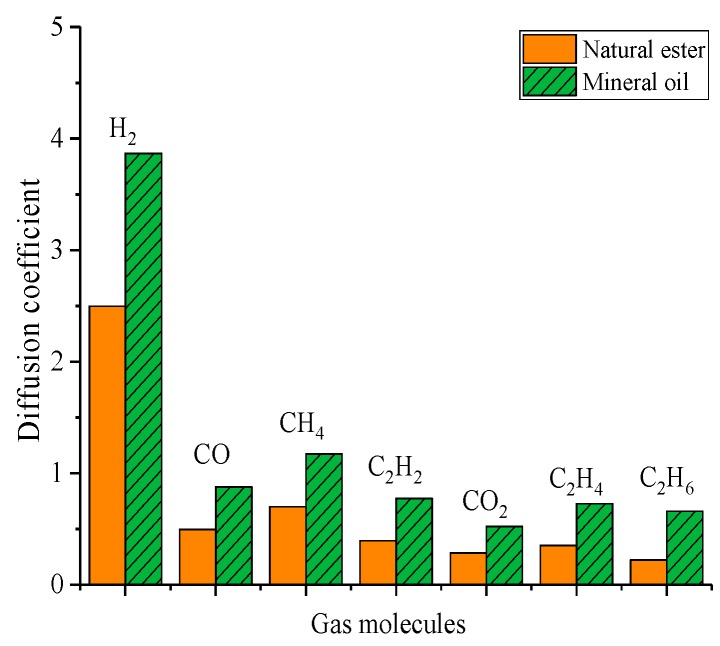
Comparison diagram of the diffusion coefficient of gas molecules in oil.

The diffusion coefficient could be used to characterize the diffusion rate of gas molecules. The diffusion coefficient of gas molecules is shown in Table 8 and Table 9. The fitting correlations in Table 8 and Table 9 were all greater than 0.95, and the simulation fitting results were reliable. The order of the diffusion coefficients of gas molecules in natural ester was as follows: H_2_ > CH_4_ > CO> C_2_H_2_ > C_2_H_4_ > CO_2_ > C_2_H_6_ and that in mineral oil was as follows: H_2_> CH_4_ > CO > C_2_H_2_ > C_2_H_4_ > C_2_H_6_ > CO_2_. All gas molecules in two oils had the same diffusion coefficient except for CO_2_. In addition, as shown in Figure 10, another obvious rule was that the diffusion coefficient of gas molecules in natural ester was less than that in mineral oil, which was consistent with the above analysis of influence law.

To prove how the three factors affect the diffusion of gas molecules, the Pearson correlation coefficient was used to calculate the correlation of the *FFV*, interaction energy, and relative molecular mass. Pearson correlation coefficients were calculated for three influencing factors and diffusion coefficients, respectively, and the results are shown in Table 10. *D*-*E*, *D*-*FFV* and *D*-*R* are the Pearson coefficients of diffusion coefficients and interaction energy, free volume, and relative molecular weight, respectively. The three factors were highly linearly correlated with the diffusion coefficient in natural ester and mineral oil. However, the interaction energy was the most important factor in natural ester, while the *FFV* was the most important factor in mineral oil.

In summary, the diffusion behavior of gas molecules in different oil media was different. The viscosity of natural ester was much higher than that of mineral oil, so the fractional free volume of gas molecules in natural ester oil was small the natural esters had higher interaction energy and stronger binding effect on gas molecules, which made the diffusion of gas molecules in natural ester hindered greatly, and the diffusion coefficient was small. In addition, a significant difference in composition between the natural ester and mineral oil resulted in different interaction energy between gas molecules and oil medium, which led to the different sequences of diffusion coefficients of gas molecules in natural ester and mineral oil.

### 3.4. Influence of Temperature on the Diffusion Behavior of Gases

In addition to the influence factors, such as the type of gas molecules and oil medium, temperature also had a great influence on the diffusion behavior of gas molecules in the oil medium. Due to the wide application of transformers, taking into account the different operating locations of different transformers and the different operating loads, the operating temperature would also vary greatly. In this work, the diffusion behaviors of gas molecules in natural ester and mineral oil at 283 K, 303 K, 323 K, 343 K, and 363 K temperatures were, respectively, simulated.

**Figure 11:**
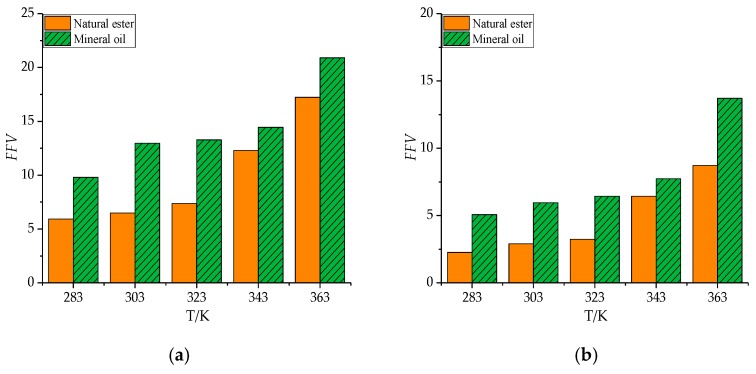

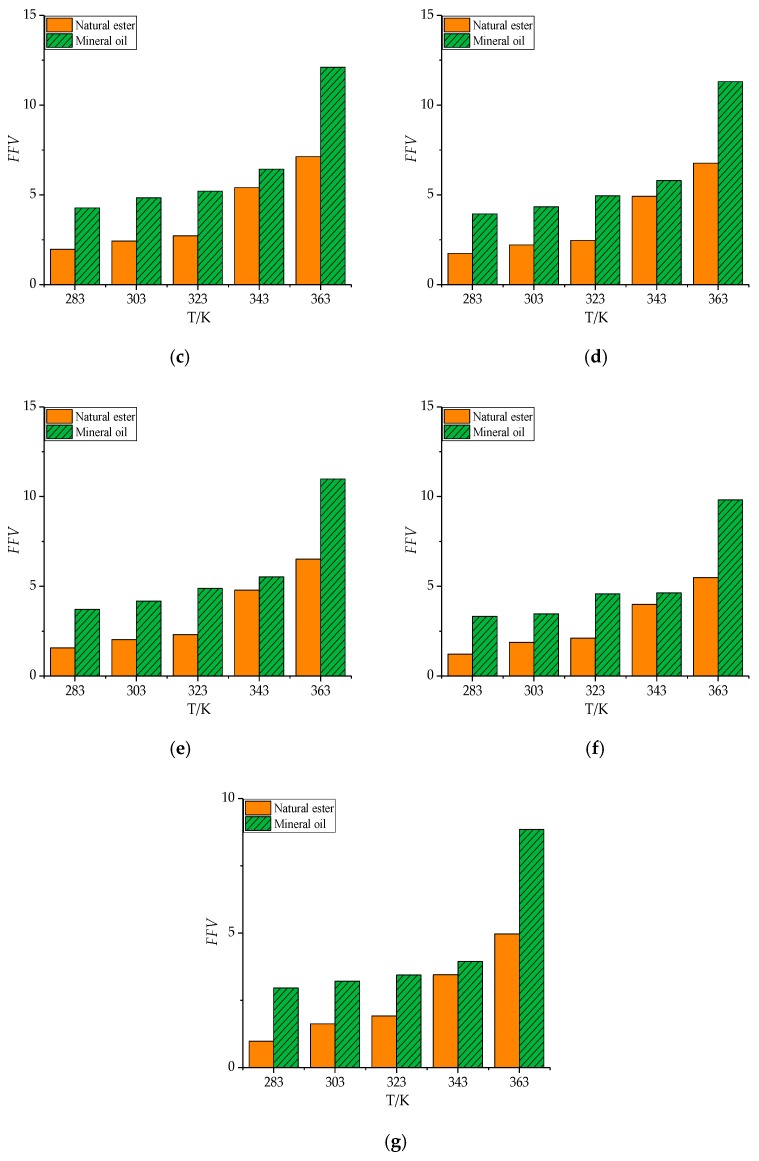
The free volume of a gas molecule varies with temperature. (**a**) H_2_, (**b**) CO, (**c**) CO_2_, (**d**) CH_4_, (**e**) C_2_H_2_, (**f**) C_2_H_4_, and (**g**) C_2_H_6_.

**Figure 12:**
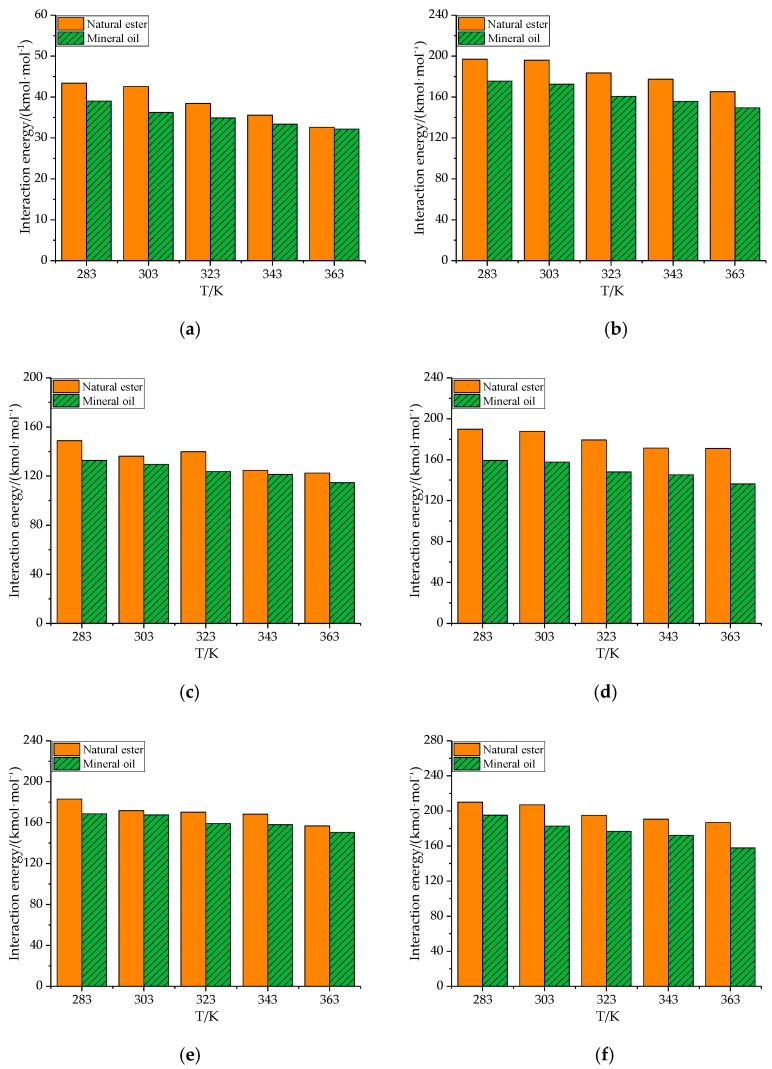

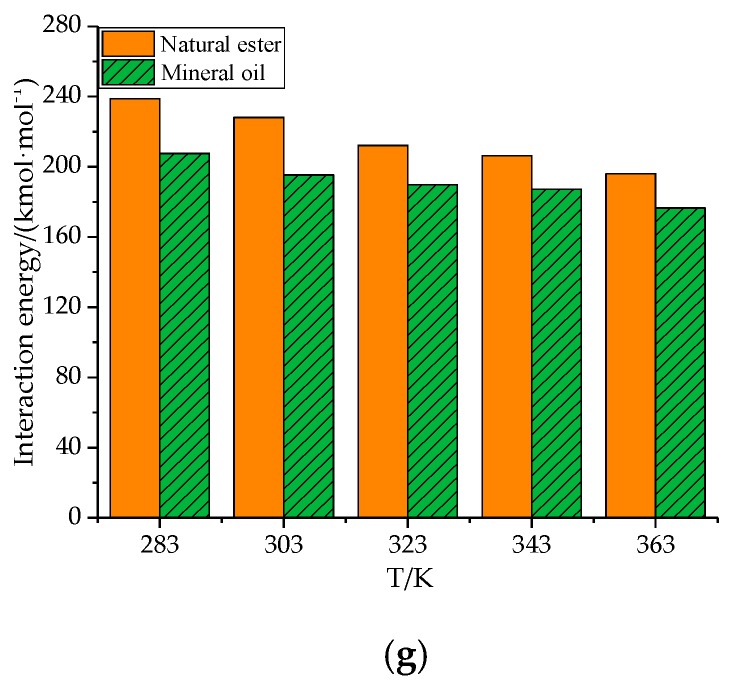
The interaction energy between the gas molecule and oil varies with temperature. (**a**) H_2_, (**b**) CO, (**c**) CO_2_, (**d**) CH_4_, (**e**) C_2_H_2_, (**f**) C_2_H_4_, and (**g**) C_2_H_6_.

The free volume of gas molecules in natural esters and mineral oils varied with temperature, as shown in Figure 11, Table 11, and Table 12. Obviously enough, *FFV* of all gas molecules in natural ester and mineral oil had increased gradually from 283 K to 363 K. There were two reasons for this phenomenon. Firstly, as the temperature increased, the volume of the model expanded, and the gas had more free space. Secondly, the higher the temperature, the lower the viscosity of the oil medium, and the larger the free volume of the gas molecules. As can be seen from Figure 12, Table 13, and Table 14, the interaction energy between gas molecules and oil had declined with increasing temperature. This phenomenon could be attributed to the increase in the kinetic energy of the gas molecules as the temperature increased. In brief, the temperature didn’t affect the ordering of free volume and interaction energy of gas molecules in natural ester and mineral oil.

The diffusion coefficients of gas molecules at different temperatures are shown in Table 15 and Table 16. It could be seen that the diffusion coefficient sequence of gas molecules in mineral oil and natural ester did not change at all temperatures, and the diffusion coefficient of gas molecules increased with the increase of temperature, which is supported by the above analysis.

**Figure 13:**
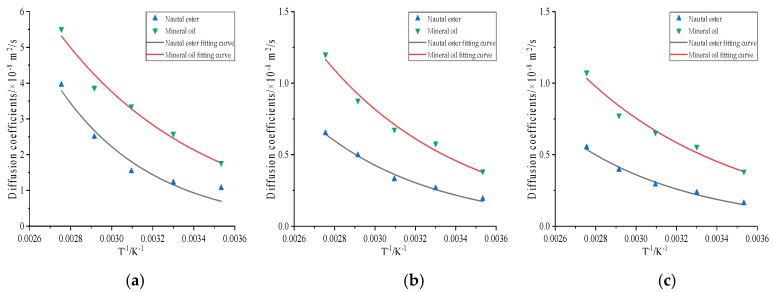
The fitting curve of gas molecular diffusion coefficient with temperature (**a**) H_2_, (**b**) CO, (**c**) C_2_H_2_.

The thermodynamic process of gas molecular diffusion follows the Arrhenius equation according to the thermodynamic formula [21], as shown in Equation (10):

(10) $$D=D_{0}e^{-E_{D}/RT}$$

where *D* is the diffusion coefficient at T temperature, *D_0_* is the preexponential factor, *E_D_* is the diffusion activation energy, and R is a constant. Thereby, Equation (8) can be simplified into the following equation:

(11) $$D=ae^{b/T}$$

where *a* and *b* are the constants, the fitting curve and equation of each gas at species temperature were fitted according to Equation (11). In Figure 13 and Table 17 and Table 18, the resulting fit curve is given, and the fitting formulas are shown. Table 17 and Table 18 provide a phenomenon that the diffusion coefficient of gas molecules had a good fitting relationship with temperature, proving the accuracy of molecular simulation.

## 4. Conclusions

The diffusion behavior of seven gas molecules in natural ester and mineral oil was studied based on molecular dynamics simulation. The fractional free volume, interaction energy, diffusion displacement, trajectory, MSD, and diffusion coefficient were analyzed. The conclusions are as follows:

The fractional free volume of gas molecules in oil was one of the important factors affecting the diffusion of gas molecules. The *FFV* order of seven gas molecules in natural ester and mineral oil was as follows: H_2_ > CO > CH_4_ > C_2_H_2_ > CO_2_ > C_2_H_4_ > C_2_H_6_. The *FFV* of gas molecules in natural ester was smaller than that in mineral oil, which was attributed to the higher viscosity of natural esters, resulting in the smaller free volume of gas molecules in natural esters.

The interaction energy between gas molecules and oil affected the diffusion behavior of gas molecules. The interaction energy between the gas molecules and two kinds of oil was the binding energy, the Van der Waals interaction energy was the main interaction energy, and the electrostatic interaction energy was extremely low. The interaction energy between gas molecules and natural ester was as following: C_2_H_6_ > C_2_H_4_ > CO > CO_2_ > C_2_H_2_ > CH_4_ > H_2_. The interaction energy between gas molecules and mineral oil was as following: C_2_H_6_ > C_2_H_4_ > CO_2_ > CO > C_2_H_2_ > CH_4_ > H_2_. The difference in interaction energy between the natural ester and mineral oil depended on the composition of the two oils.

The relative molecular weight of the gas was also a factor that influenced the diffusion behavior of gas molecules. The larger the molecular weight of gas molecules with similar structures, the greater the interaction energy. But CO and CO_2_ in natural esters were an exception.

The displacement of the gas molecules in the mineral oil was zigzag, indicating that the gas molecules diffuse in the form of continuous jumps and gas molecules could conduct continuous transformational diffusion in the holes of the medium, whether in natural ester or mineral oil. H_2_ had the greatest displacement distance. H_2_ jumped a lot more each time than other gas molecules, making it easier to spread. Compared with the diffuse displacement and trajectory of gas molecules in mineral oil, gas molecules moved closer and were more difficult to diffuse in natural ester.

The order of the diffusion coefficients of gas molecules in natural ester was as follows: H_2_ > CH_4_ > CO > C_2_H_2_ > C_2_H_4_ > CO_2_ > C_2_H_6_ and that in mineral oil was as follows: H_2_ > CH_4_ > CO > C_2_H_2_ > C_2_H_4_ > C_2_H_6_ > CO_2_. The diffusion coefficient of gas molecules in natural ester was less than that in mineral oil.

The rising temperature could enhance the free volume of gas molecules, reduce the interaction energy between gas molecules and oil, and increase the diffusion coefficient of gas molecules. Instead, the temperature didn’t affect the ordering of diffusion coefficient, free volume, and interaction energy of gas molecules in natural ester.

The diffusion of gas molecules in polymers was mainly due to the free volume, interaction energy, and relative molecular weight of gas molecules. The binding energy between gas molecules and polymers was very large, which means that polymers had a strong binding ability to gas molecules. The free volume of the gas molecules in the polymer was very small, which means there was less space for the gas molecules to spread freely. The larger the interaction energy, the smaller the free volume, and the larger the relative molecular weight of the gas, the less the diffusion of the gas.

## Figures and Tables

**Table 1:** Fatty acid compositions of natural ester.

Saturated Fatty Acid	Unsaturated Fatty Acid	Polyunsaturated Fatty Acids	
		Diunsaturated Fatty Acid	Triunsaturated Fatty Acid
16 wt.%	24 wt.%	50 wt.%	10 wt.%

**Table 2:** Compositions of mineral oil.

Chain Hydrocarbons	Alkanes			
	A Cycloalkane	Bicyclic Alkanes	Tricyclic Alkanes	Tetracyclic Alkanes
11.6 wt.%	15.5 wt.%	28.5 wt.%	23.3 wt.%	9.7 wt.%

**Table 3:** *FFV* (fraction of free volume) of gas molecules in natural ester at 343 K.

	H_2_	CO	CH_4_	C_2_H_2_	CO_2_	C_2_H_4_	C_2_H_6_
*V_o_* (Å^3^)	19463	20638	20867	20972	21003	21175	21296
*V_f_* (Å^3^)	2711	1419	1190	1085	1054	882	761
*FFV* (%)	12.29	6.43	5.40	4.92	4.78	3.99	3.45

**Table 4:** *FFV* of gas molecules in mineral oil at 343 K.

	H_2_	CO	CH_4_	C_2_H_2_	CO_2_	C_2_H_4_	C_2_H_6_
*V_o_* (Å^3^)	15253	16448	16681	16793	16842	17002	17125
*V_f_* (Å^3^)	2574.67	1379.23	1146.38	1034.11	985.46	825.37	701.84
*FFV* (%)	14.44	7.74	6.43	5.80	5.53	4.63	3.94

**Table 5:** The average interaction energy between gas molecules and natural ester at 343 K.

	H_2_	CO	CH_4_	C_2_H_2_	CO_2_	C_2_H_4_	C_2_H_6_
*E_int_* (kcal/mol)	−35.56	−177.51	−124.70	−171.39	−168.34	−190.66	−206.34
*E_vdw_* (kcal/mol)	−35.56	−176.38	−124.55	−166.53	−67.06	−187.12	−205.98
*E_elec_* (kcal/mol)	0	−1.13	−0.15	−4.86	−1.28	−3.54	−0.36

**Table 6:** The average interaction energy between gas molecules and mineral oil at 343 K.

	H_2_	CO	CH_4_	C_2_H_2_	CO_2_	C_2_H_4_	C_2_H_6_
*E_int_* (kcal/mol)	−33.36	−155.80	−121.31	−145.21	−158.10	−172.27	−187.26
*E_vdw_* (kcal/mol)	−33.36	−155.72	−121.23	−144.48	−158.06	−171.82	−187.1
*E_elec_* (kcal/mol)	0	−0.08	−0.07	−0.73	−0.04	−0.45	−0.16

**Table 7:** The relative molecular mass of gas molecules.

	H_2_	CO	CH_4_	C_2_H_2_	CO_2_	C_2_H_4_	C_2_H_6_
Relative molecular mass	2	30	16	30	48	32	34

**Table 8:** Diffusion coefficients of seven gas molecules in natural ester at 343 K.

	H_2_	CO	CH_4_	C_2_H_2_	CO_2_	C_2_H_4_	C_2_H_6_
Slope a	14.9801	2.9812	4.2021	2.3690	1.7128	2.1208	1.3335
Fitting correlation coefficient R^2^	0.99	0.98	0.99	0.99	0.97	0.99	0.98
Diffusion coefficient D/×10^−8^ m^2^/s	2.4967	0.4969	0.7003	0.3948	0.2855	0.3535	0.2223

**Table 9:** Diffusion coefficients of seven gas molecules in mineral oil at 343 K.

	H_2_	CO	CH_4_	C_2_H_2_	CO_2_	C_2_H_4_	C_2_H_6_
Slope a	23.2031	5.2672	7.0346	4.6477	3.1398	4.3617	3.9591
Fitting correlation coefficient R^2^	0.99	0.99	0.98	0.99	0.99	0.99	0.97
Diffusion coefficient D/×10^−8^ m^2^/s	3.8677	0.8779	1.1724	0.7746	0.5233	0.7270	0.6598

**Table 10:** Pearson correlation coefficient in oil.

	*D-E*	*D-FFV*	*D-R*
Natural ester	0.9793	0.9558	−0.8554
Mineral oil	0.9488	0.9543	−0.8499

**Table 11:** *FFV* of gas molecules in natural ester at different temperatures.

	*FFV*/%						
T/K	H_2_	CO	CH_4_	C_2_H_2_	CO_2_	C_2_H_4_	C_2_H_6_
283 K	5.93	2.26	197	1.74	1.56	1.22	0.98
303 K	6.48	2.90	2.43	2.21	2.03	1.87	1.63
323 K	7.37	3.24	2.72	2.46	2.31	2.11	1.92
343 K	12.29	6.43	5.40	4.92	4.78	3.99	3.45
363 K	17.22	8.72	7.13	6.77	6.52	5.48	4.97

**Table 12:** *FFV* of gas molecules in mineral oil at different temperatures.

	*FFV*/%						
T/K	H_2_	CO	CH_4_	C_2_H_2_	CO_2_	C_2_H_4_	C_2_H_6_
283 K	9.79	5.07	4.27	3.94	3.71	3.32	2.96
303 K	12.97	5.95	4.84	4.34	4.17	3.46	3.21
323 K	13.28	6.43	5.21	4.95	4.88	4.58	3.44
343 K	14.44	7.74	6.43	5.80	5.53	4.63	3.94
363 K	20.90	13.71	12.11	11.31	10.98	9.81	8.85

**Table 13:** The interaction energy between gas molecules and mineral oil at different temperatures.

	*E_int_*/(kcal/mol)						
T/K	H_2_	CO	CH_4_	C_2_H_2_	CO_2_	C_2_H_4_	C_2_H_6_
283 K	−43.38	−196.98	−148.83	−189.77	−183.04	−210.01	238.86
303 K	−42.56	−196.00	−136.14	−187.69	−171.69	−207.08	228.09
323 K	−38.45	−183.55	−139.87	−179.22	−170.23	−195.12	−212.16
343 K	−35.56	−177.51	−124.70	−171.39	−168.34	−190.66	−206.34
363 K	−32.61	−165.22	−122.43	−170.94	−156.82	−186.68	−195.96

**Table 14:** The interaction energy between gas molecules and mineral oil at different temperatures.

	*E_int_*/(kcal/mol)						
T/K	H_2_	CO	CH_4_	C_2_H_2_	CO_2_	C_2_H_4_	C_2_H_6_
283 K	−39.01	−175.62	−132.75	−159.30	−168.59	−195.16	−207.63
303 K	−36.25	−172.51	−129.59	−157.70	−167.48	−182.73	−195.24
323 K	−34.91	−160.68	−123.69	−147.93	−159.18	−176.81	−189.75
343 K	−33.36	−155.80	−121.31	−145.21	−158.10	−172.27	−187.26
363 K	−32.17	−149.44	−114.72	−136.42	−150.58	−157.78	−176.52

**Table 15:** Diffusion coefficients of gas molecules in natural ester at different temperatures.

	Diffusion Coefficients *D*/×10^−8^ m^2^/s						
T/K	H_2_	CO	CH_4_	C_2_H_2_	CO_2_	C_2_H_4_	C_2_H_6_
283 K	1.0657	0.1915	0.2943	0.1613	0.1227	0.1457	0.1216
303 K	1.2222	0.2652	0.3592	0.2349	0.2005	0.2161	0.1981
323 K	1.5351	0.3296	0.4631	0.2916	0.2582	0.2689	0.2167
343 K	2.4967	0.4969	0.7003	0.3948	0.2855	0.3535	0.2723
363 K	3.9543	0.6503	1.0189	0.5512	0.4664	0.5462	0.4004

**Table 16:** Diffusion coefficients of gas molecules in mineral oil at different temperatures.

	Diffusion Coefficients *D*/×10^−8^ m^2^/s						
T/K	H_2_	CO	CH_4_	C_2_H_2_	CO_2_	C_2_H_4_	C_2_H_6_
283 K	1.7678	0.3827	0.5849	0.3819	0.2834	0.3042	0.2927
303 K	2.5852	0.5779	0.6082	0.5558	0.4288	0.4933	0.4594
323 K	3.3513	0.6750	0.8987	0.6554	0.4599	0.5711	0.5358
343 K	3.8677	0.8779	1.1724	0.7746	0.5233	0.7270	0.6598
363 K	5.5090	1.2009	1.4043	1.0747	0.6264	0.8890	0.8664

**Table 17:** The fitting formula of the diffusion coefficient and temperature in natural ester.

Gas Molecules	Fitting Formula	R^2^
H_2_	*D* = 1516.15487*e*^−2174.98083*/T*^	0.95
CO	*D* = 67.01119*e*^−1685.98837*/T*^	0.98
CH_4_	*D* = 177.88902*e*^−1885.69621*/T*^	0.97
C_2_H_2_	*D* = 47.60204*e*^−1628.25238*/T*^	0.98
CO_2_	*D* = 41.14987*e*^−1647.59101*/T*^	0.95
C_2_H_4_	*D* = 72.30426*e*^−1790.25078*/T*^	0.97
C_2_H_6_	*D* = 21.70519*e*^−1467.3526*/T*^	0.95

**Table 18:** The fitting formula of the diffusion coefficient and temperature in mineral oil.

Gas Molecules	Fitting Formula	R^2^
H_2_	*D* = 262.1956*e*^−1415.01326*/T*^	0.97
CO	*D* = 63.09076*e*^−1448.72382*/T*^	0.98
CH_4_	*D* = 50.27274*e*^−1297.76786*/T*^	0.97
C_2_H_2_	*D* = 35.09568*e*^−1279.70589*/T*^	0.97
CO_2_	*D* = 6.99573*e*^−878.38744*/T*^	0.95
C_2_H_4_	*D* = 27.95973*e*^−1251.07713*/T*^	0.98
C_2_H_6_	*D* = 30.18511*e*^−1296.12329*/T*^	0.98
